# Effect of adding short foot exercise to hip and knee focused exercises in treatment of patients with patellofemoral pain syndrome: a randomized controlled trial

**DOI:** 10.1186/s13018-024-04688-x

**Published:** 2024-04-01

**Authors:** Abdallah Mohamed kamel, Karim Ghuiba, Dina S Abd Allah, Nadia Abdelazem Fayaz, Nasr Awad Abdelkader

**Affiliations:** Department of Physical Therapy for Musculoskeletal disorders and its Surgeries, Faculty of Physical Therapy, Cairo University, Giza, Egypt

**Keywords:** Anterior knee pain, Patellofemoral pain syndrome, Short foot exercise

## Abstract

**Background:**

Patellofemoral pain syndrome is considered a common cause of anterior knee pain that could disturb function and limit daily activities. The purpose of the study was to investigate the effect of adding short foot exercise on pain, function, balance, and hip abductors, and quadriceps muscles strength in the treatment of patients with patellofemoral pain syndrome.

**Methods:**

Twenty-eight male and female patients with patellofemoral pain syndrome with age ranged from 18 to 35 years old participated in this study. They were equally and randomly assigned into two groups; the study group which received short foot exercise in addition to hip and knee exercises (*n* = 14) and thecontrol group which received hip and knee exercises only (*n* = 14). Participants received their interventions during 6 consecutive weeks (12 sessions). Pain intensity, function, abductors quadriceps muscle strength, and balance were assessed using the Visual Analog Scale, anterior knee pain scale (AKPS), hand-held dynamometer, and the Biodex Balance System respectively. All measurements were taken before and after 6 weeks of intervention in both groups. Multivariate analysis of variance was performed to compare the within and between groups effects for measured variables.

**Results:**

The within-group comparison showed significant improvement in pain severity, function, balance, and hip abductors, and quadriceps muscles strength in both groups post-treatment compared with pre-treatment. Between groups analysis, however, showed no significant statistical difference between both groups in all variables, except in pain, function, and mediolateral stability which showed better improvement compared to the control group.

**Conclusions:**

Adding short foot exercise to hip and knee exercises improved pain, function, and mediolateral stability in patients with patellofemoral pain syndrome.

**Trial registration:**

clinicaltrials.gov. NO: NCT05383781. Date 19/ 5/2022.

**Supplementary Information:**

The online version contains supplementary material available at 10.1186/s13018-024-04688-x.

## Background

Patellofemoral pain syndrome (PFPS) is one of the most common knee problems that disturb function and daily activities [1]. Symptoms of PFPS can develop either slowly or abruptly, and pain tends to worsen with activities such as squatting, prolonged sitting, stair climbing, jumping, or running [2]. PFPS affects approximately 25% of physically active individuals [3]. The development of patellofemoral pain is believed to have multiple contributing factors, including proximal, local, and distal factors.

Distal factors, such as abnormal foot posture and mobility, including excessive mid-foot mobility, prolonged rear foot pronation, and increased navicular drop values during stance, have been associated with increased stresses on the lateral patellofemoral joint and the development of PFPS [4–6]. Excessive foot eversion during the stance phase of gait could increase tibial internal rotation concerning the talus, which in turn increases hip internal rotation, hip adduction, and the dynamic quadriceps angle [7]. Such abnormal kinematics could increase compression over the lateral patellofemoral joint (PFJ) [8].

Previously, it was believed that PFPS was a self-limiting disorder that mainly affected adolescents and would resolve over time [9]. Unfortunately, the long-term prognosis for this condition is relatively poor, with only about one-third of cases fully recovering and about one-fourth of individuals having to limit their activities or stop participating in sports due to persistent pain [3, 10]. Despite the high prevalence of patellofemoral pain (PFP) and positive short-term treatment outcomes, approximately 80% of those who completed rehabilitation programs still experience pain. Additionally, at a 5-year follow-up, 74% of individuals had to reduce their physical activity [11–14]. These outcomes have a negative impact on their quality of life, leading to limited physical activity, loss of self-identity, and pain-related psychological and emotional disorders such as confusion, fear, and potentially even depression [15].

Given the high failure rate of conventional treatment (hip and knee muscles strengthening, foot orthoses, and patellar taping) for patellofemoral pain, there is a need for a paradigm shift in identifying subgroups of individuals with this condition and providing appropriate stratified care [4, 16, 17].

Selfe et al. started this process by identifying three distinct subgroups of patients with patellofemoral pain: the first group was a strong group that had the greatest rectus femoris length, function, quality of life, and lowest pain scores; the second group was a weaker and tighter group who had a higher body mass index (BMI) and a longer duration of pain; and the last group was a weak and pronated foot group who had greater patellar mobility and a shorter duration of pain. However, further research and intervention studies are necessary to investigate patient outcomes and determine the most effective treatments for each subgroup [18].

Research regarding the impact of foot pronation on PFP has primarily focused on the recommendation of foot orthoses. However, foot orthoses alone may not be sufficient for all patients, as they are passive methods of treatment [10]. Current rehabilitation approaches emphasize active participation from the patient, especially when addressing intrinsic foot muscle weakness in conjunction with a pronated foot posture [14, 19]. To strengthen the intrinsic foot muscles, reduce foot pronation, and enhance the medial longitudinal arch (MLA), several active exercises can be employed. These exercises include toe curls and the short foot exercise (SFE) [20, 21].

The SFE is commonly prescribed in sports and rehabilitation settings and focuses on strengthening the intrinsic foot muscles and improving the longitudinal and transverse arches. Addressing the foot with SFE may be helpful to improve treatment outcomes and decrease the recurrence rate of PFPS. This study was the first to use the objective method of the Biodex Balance System to assess balance at PFPS patients .Also, this study tried to build up growing evidence for adding SFE to the standard treatment for PFPS patients because there was a limited number of studies addressing this point, and hence would be added to the American Physical Therapy Association Guidelines for PFPS management. Therefore, the purpose of the study was to investigate the effect of adding SFE to hip and knee focused exercises on pain, function, balance, hip abductors, and quadriceps muscles strength in the treatment of patients with patellofemoral pain syndrome.

## Methods

### Study design

This randomized controlled trial was conducted at the outpatient clinic of the Faculty of Physical Therapy, Cairo University between May 2022 and August 2023. The study was approved by the ethical committee of the same institution, approval number (P.T.REC/012/003641). The procedure and purposes of the study were explained to each participant before starting the study. Then, each participant was asked to sign a consent form. They were randomly assigned into two equal groups: The study group (*n* = 14) received hip and knee focused exercises in addition to short foot exercise, and the control group (*n* = 14) received hip and knee focused exercises only.

### Participants

Individuals aged 18–35 years old [22] with a BMI ranging from 18 to 25 kg/m^2^ [23] were eligible for enrollment. For patients who had (1) anterior or retropatellar knee pain for at least six weeks [22], (2) navicular drop test of more than 10 mm [24], and (3) pain elicited at least by two of the following four tests: (i) isometric muscle contraction with a slight bent knee, (ii) palpation of the patellofemoral joint line, (iii) patellar compression against the femoral bone, and (iv) active resisted knee extension were enrolled in the study [25]. Patients who (1) underwent previous knee surgery [25], (2) had knee pathologies (e.g., osteoarthritis, patellar tendinopathy, lesions of menisci, cartilage, bone, or ligaments) [25], (3) underwent physical therapy 4 weeks before enrollment in the study [26], (4) had taken non-steroidal anti-inflammatory drugs during the previous week [26], and (5) psychological disorders [25] were excluded from the study.

### Randomization and blinding

Randomization was generated by a random number generator with an allocation ratio of 1:1 using the website http://www.randomization.com. The statistical analyzer and participants were blinded. Before the initiation of the study, sequentially numbered sealed opaque envelopes were used to conceal the allocations. All participants were blinded to group allocation by ensuring that they were unaware of the exercises performed by the other group. To maintain the blinding, the intervention sessions were delivered separately to members of each treatment group.

### Outcome measures

The first primary outcome measure was pain measured by the visual analogue scale (VAS), which is a well-known, valid, and reliable measuring tool for pain [22, 23]. The VAS consists of a 10-centimeter line where the patient rates their pain, ranging from 0 (indicating no pain) to 10 (representing the worst pain imaginable [27, 28]. The second primary outcome measure was knee function measured by the anterior knee pain scale (AKPS)(Kujala), which is a valid and reliable tool [13, 24] and the gold standard for evaluating knee function in PFPS [25]. It is a weighted questionnaire that covers 13 different areas, including pain and functionality. Specifically, it assesses responses to six activities related to AKPS, such as walking, running, jumping, climbing stairs, squatting, and sitting for prolonged periods with the knee bent [29]. The secondary outcome measures, balance and muscle strength, which were measured by the Biodex Balance System (BBS) and hand-held dynamometer (HHD), respectively. The BBS is a valid and reliable system for measuring balance [30], while the HHD is valid and reliable for measuring muscle strength [31]. The strength of the knee extensor (quadriceps) was evaluated while the participants were sitting on the examination table with the hip in 90° flexion, the knee in 60° flexion, and their arms held against their chests. The HHD was positioned near the malleoli, while for hip abductors, the participants were positioned in a side-lying position with the evaluated limb in a neutral position with a pillow between the legs and the dynamometer placed over the lateral femoral condyle. A familiarization trial was done, then three test trials were done, and the mean was calculated and recorded [32]. For balance assessment, the ability to stand on one leg (single-leg balance) using the BBS device was evaluated. Initially, each participant was allowed to become accustomed to the device. The participant was instructed to stand on the platform with their experimental side (unilateral standing), barefooted, and with their hands at their sides while maintaining an extended knee. The stability platform was then unlocked, allowing movement. The participant was instructed to adjust their foot position until they could maintain a moving point in the center or near the center of the circles for 20 s. The stability level was set at 8. Then, the participant’s balance was assessed while standing on the experimental limb, and the stability index was recorded. The platform was locked, and the placement of the participant’s feet was saved and documented. To obtain balance indices for each patient, they were asked to complete a familiarization trial followed by three test trials, keeping their eyes open. To consider a complete trial, the patient must maintain balance for 20 s. Then the means of the three test tails were calculated and recorded [33]. The stability indices that were recorded were the overall stability index (OASI), mediolateral stability index (MLSI), and anterior-posterior stability index (APSI) represent the variance of foot platform displacement in degrees, in all motions, in the sagittal plane and the frontal plane, respectively. The patient’s score on this test assesses deviations from the center, thus a lower score indicates a better balance [34]. Each patient was assessed before starting the first session and after receiving the last session of treatment.

### Intervention

The participants in the study group received the SFE program. To perform SFE, participants were A to elevate the MLA, shorten the foot in the anterior-posterior line, and approximate the first metatarsal head toward the heel without toe flexion. The elevated MLA position would be maintained for five seconds in each repetition. Participants performed the SFE in 3 sets of 15 repetitions each day for two days per week for 6 weeks (with at least one day between each session) [24]. The participants had to start the exercise in a sitting position (in the first and second weeks) and then progress to a double stance (in the third and fourth weeks), then a single-leg stance position (in the fifth and sixth weeks) [24, 35]. In addition to the SFE program, the participants in this group received hip and knee focused exercise program, while the participants in the control group received only a hip and knee focused exercise program.

### Hip-focused exercise

The hip-focused exercises were based on previous studies and consisted of side-lying hip abduction, hip external rotation (clamshell), and prone hip extension [36, 37].

### Knee-focused exercise

The knee-focused exercise regime was based on previous studies [34, 35] and consisted of supine straight leg raises, supine terminal knee extensions (from 10° flexion to full extension), and a mini-squat (45° flexion) with the back supported against the wall (to reduce stabilizing requirements from the hip muscles) [38]. For the hip and knee focused exercises: the number of repetitions is increased from 3 sets of 10 repetitions to a maximum of 3 sets of 20 repetitions.Thereafter resistance is increased using a weight cuff or resistance tubing. Repetitions were performed dynamically over 2–3 s. 2-second pause between repetitions. 30-second pause between sets for two days per week for six weeks. Minimum one rest day between sessions [24]. Details of the exercise program are provided in the appendix.

### Statistical analysis

A sample size calculation (G Power 3.1.9.7) based on the pain intensity (using two-tailed α:0.05, β:0.20 (power: 80%)) was conducted to detect a mean difference of 20 points on a 0-100 numerical pain rating scale [39], considering an effect size=1.33   [25]. Then we increased about 10% of the estimated number to ensure adequate power. It was determined that 11 participants would be required for each group. We increased 25% to overcome the expected dropouts. The total number was 14 for each group. The total sample size was 28 subjects. Data were expressed as mean ± SD. Shapiro-Wilk and Kolmogrov-Smirnov tests were used for testing the normality of data distribution and showed that all measured variables were normally distributed. Unpaired t-tests and chi-square were used to compare the subjects’ characteristics of the two groups. A Multivariate analysis of variance (MANOVA) was performed to compare within and between groups’ effects for measured variables. A statistical package for the social sciences computer program (version 20 for Windows; SPSS Inc., Chicago, Illinois, USA) was used for data analysis. *P* ≤ .05 was considered significant.

## Results

**Fig. 1:**
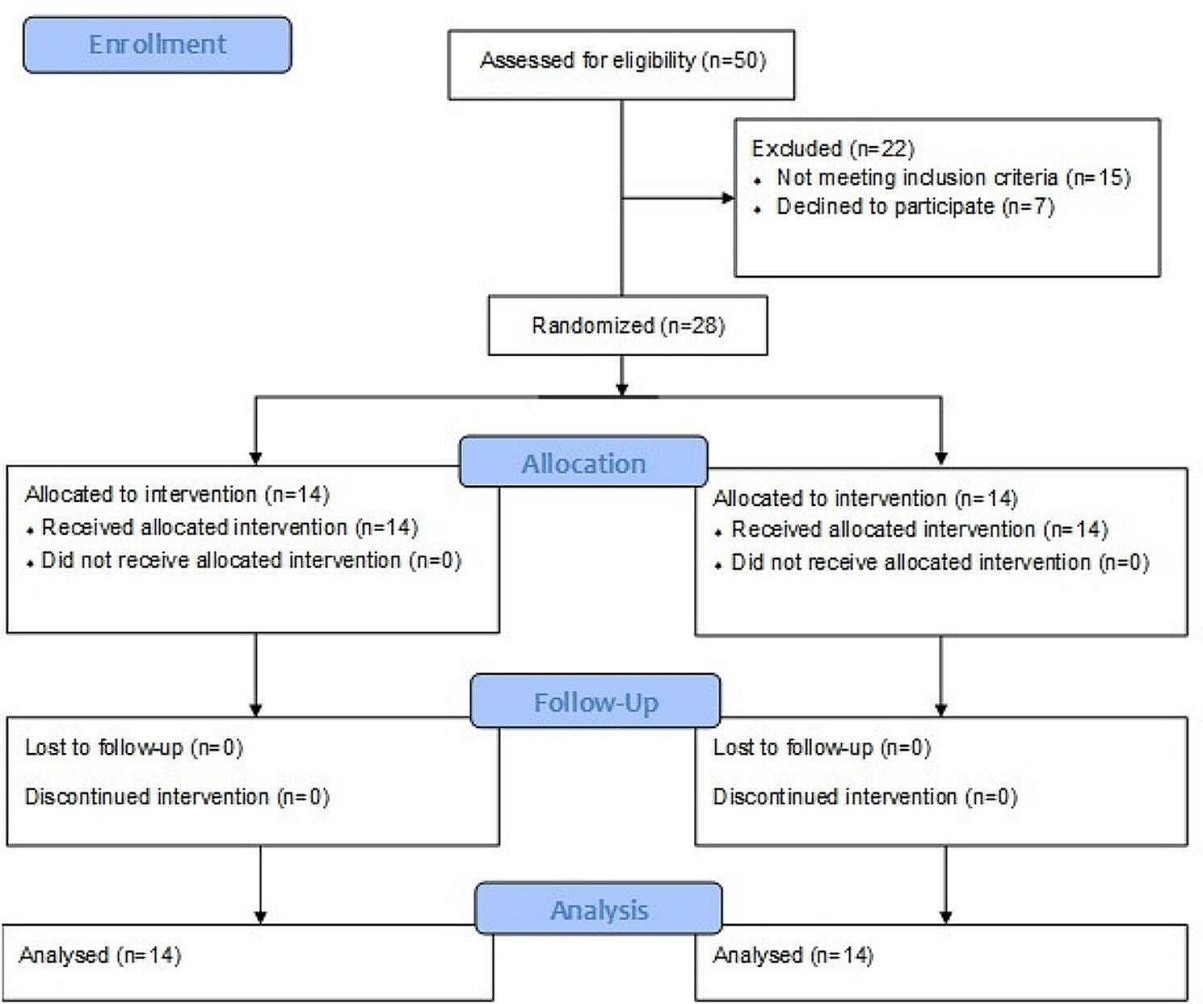
Flowchart for patients eligibility

Fifty patients were assessed for eligibility. Fifteen did not meet the inclusion criteria and 7 refused to join the study (Fig. 1). The study population consisted of 21 men and 7 women aged 18–35 years old with PFPS who were randomly assigned to 2 equal groups. The 2 groups were comparable with no significant difference in any of the demographic characteristics (Table 1).

**Table 1:** Demographic data of subjects of both groups

Demographic data	Study group	Control group	*p*-value
Age (years)	21.3 ± 1.3	22.5 ± 3	0.211
Weight (kg)	71.9 ± 12.4	70.7 ± 13.7	0.808
Height (cm)	171 ± 12	170 ± 11.7	0.801
BMI (Kg/m^2^)	24.4 ± 2.25	24.2 ± 2.74	0.870

Abbreviation: BMI, body mass index

### Post-treatment changes

The mean values of the VAS score in both groups before treatment (Table 2) were comparable (MANOVA, *P* = .284). MANOVA revealed significant within-group effects *(P* = .001) and between-group effects *(P* = .001). The VAS scores improved significantly in both groups after treatment *(P* = .001). The improvements in the study group were better than those in the control group. The difference between groups after treatment was statistically significant (*P* = .001).

**Table 2:** Mean ± SD of outcomes pre- and post-treatment of both groups

**Measured variables**	**Control group**	**Study group**	***p*** **-value**	**MD (95% CI) between groups**	η^**2**^
**Knee pain (cm)**					
Pre-treatment	6.2 ± 1	5.8 ± 1.1	0.284	0.4 (-0.37, 1.2)	0.04
Post-treatment	3.3 ± 1.2	0.7 ± 0.6	0.001^*^	2.6 (1.8, 3.4)	0.64
*P*-value	0.001^*^	0.001^*^			
MD (95% CI), (Baseline – post)	2.9 (2, 3.7)	5.1 (4.3, 5.9)			
**AKPS**					
Pre-treatment	81 ± 5.3	79.6 ± 4.9	0.419	1.6 (-2.4, 5.5)	0.03
Post-treatment	92.6 ± 3.7	96.6 ± 3.9	0.010^*^	-4 (-7, -1)	0.23
*P*-value	0.001^*^	0.001^*^			
MD (95% CI), (Baseline – post)	-11.6 (-11, 8.7)	-17 (-19.6, -14.3)			
**Abductor muscles**					
Pre-treatment	116 ± 25	132 ± 39	0.211	-16 (-41, 9.7)	0.06
Post-treatment	179 ± 21	198 ± 43	0.158	-19 (-45,7.8)	0.08
*P*-value	0.001^*^	0.001^*^			
MD (95% CI), (Baseline – post)	-63 (-83, -43)	-66 (-85, -46)			
**Quadriceps muscle**					
Pre-treatment	104.2 ± 22.8	102.2 ± 15.4	0.79	2 (-13, 17)	0
Post-treatment	125.3 ± 19.8	139.4 ± 20	0.072	-14.1 (-29, 1.4)	0.12
*P*-value	0.035^*^	0.001^*^			
MD (95% CI), (Baseline – post)	-21 (-32, -9.7)	-37 (-48, -25)			
**OASI**					
Pre-treatment	2 ± 0.4	2.1 ± 0.5	0.881	-1 (-0.4, 0.4)	0
Post-treatment	1.7 ± 0.3	1.5 ± 0.2	0.093	0.2 (-0.04, 0.4)	0.11
*P*-value	0.014^*^	0.001^*^			
MD (95% CI), (Baseline – post)	0.3 (0.09, 0.67)	0.6 (0.3, 0.9)			
**APSI**					
Pre-treatment	1.6 ± 0.3	1.5 ± 0.3	0.749	0.1 (-0.2, 0.3)	0
Post-treatment	1.4 ± 0.36	1.2 ± 0.2	0.082	0.2 (-0.03, 0.5)	0.11
*P*-value	0.181	0.015^*^			
MD (95% CI), (Baseline – post)	0.2 (-0.09, 0.48)	0.3 (0.07, 0.65)			
**MLSI**					
Pre-treatment	1.4 ± 0.3	1.45 ± 0.3	0.509	-0.05 (-0.3, 0.1)	0.02
Post-treatment	1.2 ± 0.2	1 ± 0.2	0.012*	0.2 (0.05, 0.4)	0.22
*P*-value	0.001*	0.001*			
MD (95% CI), (Baseline – post)	0.2 (0.01, 0.34)	0.45 (0.3, 0.63)			

Abbreviations: AKPS: anterior knee pain scale, APSI: anteroposterior stability index, CI: confidence interval, MD: mean difference, MLSI: mediolateral stability index, η^2^: effect size; OASI: overall stability index, SD: standard deviation.

^*^ Indicates significant differences (*p* < .05)

There was no significant difference between the mean values of the AKPS score in both groups before treatment (Table 2) (*P* = .419), while there were significant within-group effects (*P* = .001) and between-group effects (*P* = .010). The AKPS scores improved significantly in both groups after treatment (*P* = .010). The improvements in the study group were better than those in the control group. The difference between groups after treatment was statistically significant (*P* = .010).

The mean values of hip abductors strength in both groups before treatment (Table 2) were also comparable (MANOVA, *P* = .211), and MANOVA revealed significant within-group effects (*P* = .001) but no significant between-group effects (*P* = .158). The hip abductors strength improved significantly in both groups after treatment (*P* = .001).

Regarding the mean values of quadriceps muscle strength in both groups before treatment, no significant difference was detected (Table 2) (*P* = .79), while there were significant within-group effects (*P* = .001) but no significant between-group effects were detected (*P* = .072). The quadriceps muscle strength improved significantly in both groups after treatment (*P* = .001 for the study group and *P* = .035 for the control group).

For the OASI score, there was no significant difference in mean values for both groups before treatment (Table 2) (*P* = .881). MANOVA revealed significant within-group effects (*P* = .001) but no significant between-group effects (*P* = .093). The OASI scores improved significantly in both groups after treatment (*P* = .001). The difference between groups after treatment was statistically insignificant (*P* = .093).

The mean values of APSI scores in both groups before treatment (Table 2) were comparable (MANOVA, *P* = .749). MANOVA revealed significant within-group effects (*P* = .015) but no significant between-group effects (*P* = .082). The APSI scores improved significantly in the study group after treatment (*P* = .015). The difference between groups after treatment was statistically insignificant (*P* = .082).

Finally, for MLSI score mean values, MANOVA revealed no significant difference in both groups before treatment (Table 2) (*P* = .509), but there were significant within-group effects (*P* = .001) and between-group effects (*P* = .012). The MLSI scores improved significantly in both groups after treatment (*P* = .001). The improvements in the study group were better than those in the control group. The difference between groups after treatment was statistically significant (*P* = .012).

## Discussion

The purpose of the study was to investigate the effect of adding SFE on pain, function, balance, hip abductors, and quadriceps muscles strength in the treatment of PFPS. The results of this study showed that there was a statistically significant decrease in pain and a significant increase in function, balance, hip abductors, and quadriceps muscles in both the control and study groups post-treatment when compared with pre-treatment, while between groups there was no significant statistical difference between both groups in all variable except pain, function, and mediolateral stability in favor of the study group. To our knowledge, this is the first study to assess the effects of SFE on balance in PFPS patients.

The results of this study come in accordance with Kısacık et al., who demonstrated that individuals with PFPS who received SFE along with hip and knee strengthening and stretching exercises experienced a greater reduction in knee pain and functional improvements compared to those who only received hip and knee exercises [24]. Similarly, Mølgaard et al., investigated the effects of adding SFE to the knee focused exercise in PFPS, and the results indicated improved knee pain following adding foot-focused exercises and foot orthoses to knee-focused exercise program [25]. This improvement may be attributed to the positive effect of SFE on correcting foot pronation, improving intrinsic muscles performance, and enhancing arch support leading to decreasing stresses on the PFJ [40, 41].

Previous studies have found that individuals with a pronated foot exhibit reduced electromyographic activity in the Abductor Hallucis (AbdH) muscle [42, 43], which works together with the peroneus longus muscle to support the MLA.The AbdH muscle plays a vital role in preventing excessive arch flattening during heel strike and in elevating the arch before toe-off during walking [44]. Another study reported a significant increase in the cross-sectional area of the AbdH muscle after performing the SFE exercises with foot orthoses [45]. This suggests that the SFE can elevate MLA through a closed kinetic chain mechanism, potentially correcting foot pronation and altering foot biomechanics [22]. Moreover, these changes in foot mechanics may have a kinematic impact on the entire lower extremity, potentially reducing internal tibial rotation and hip adduction. This, in turn, may lead to a decrease in internal femoral rotation, thereby reducing lateral compressive forces on the patella. This can be beneficial for improving knee pain [46–48].

Multiple previous studies proved the positive effects of the SFE on balance. Lee et al.found a significant improvement in the dynamic balance components (overall balance index, mediolateral balance index, and anteroposterior balance index) in patients with ankle sprains after performing the SFE [49]. Moon et al. also reported a significant improvement in anterior-posterior and medial-lateral stability in patients with a pronated foot after a program of SFE [50]. Lynn et al.demonstrated improvements in anterior-posterior and medial-lateral control of the center of pressure in the dominant lower extremity after a 4-week program of either towel curl exercise or SFE [51].

Mulligan and Cook observed an improvement in balance ability in healthy individuals after 4 weeks of SFE training [52]. These findings can be explained by the foot’s role in balance. The foot acts as a mechanical support for the body and provides sensory information about body position through plantar receptors. Changes in foot posture, sensation, flexibility, and strength can affect balance [53]. Moon et al.suggested that SFE stimulates the cutaneous receptors at the bottom of the foot, leading to increased afferent stimulation, improved stability, and voluntary muscle activity [50].

Newsham also highlighted the role of plantar intrinsic muscles in improving dynamic balance by controlling the arch position and stimulating proprioceptors on the sole [41]. Additionally, studies by Rothermel et al. and Janda and Vavrova indicate that SFE improves neuromuscular activity and stimulates the neurocircuitry in the sole , enhancing postural and core stability [54, 55]. Foot strength is believed to influence somatosensory control of standing posture and balance through its impact on muscle and tendinous receptors, including the plantar cutaneous receptors [56].

Conversely, in contrast to the findings of the current study, Kısacık et al. did not find a statistically significant difference in dynamic function and balance after adding foot core training (FCT) to exercise therapy for patients with PFPS. They attributed these results to the understanding that dynamic balance impairment in PFPS patients is influenced not only by foot pronation but also by knee biomechanics and pain. Additionally, their study focused exclusively on females, and it is known that there are differences in the development of sensory systems and balance control between females and males. They suggest that further studies tailored to males may demonstrate different outcomes. However, Kısacık et al. recommended that longer training periods and additional set-ups with more intensive dynamic components could potentially lead to improvements in dynamic function and balance when using FCT in combination with exercise therapy [57].

### Limitations

This study was conducted for a short term (six weeks) without patient follow-up to investigate the effect of adding SFE on pain, function, balance, hip abductors, and quadriceps muscles strength in the treatment of patients with PFPS.

## Conclusions

The findings of this study showed that adding SFE to hip and knee exercise improved pain, function, and mediolateral stability in the treatment of patients with PFPS. This can help clinicians improve their treatment program for PFPS patients to achieve better results with them, decrease recurrence rate, and improve disease prognosis.

### Electronic supplementary material

Below is the link to the electronic supplementary material.

Supplementary Material 1

## Data Availability

No datasets were generated or analysed during the current study.

## Abbreviations

PFPS: Patellofemoral pain syndrome
PFJ: Patellofemoral joint
PFP: Patellofemoral pain
BMI: Body mass index
MLA: Medial longitudinal arch
SFE: Short foot exercise
VAS: Visual analogue scale
AKPS: Anterior knee pain scale
BBS: Biodex balance system
HHD: Hand-held dynamometer
OASI: Overall stability index
MLSI: Mediolateral stability index
APSI: Anteroposterior stability index
AbdH: Abductor Hallucis
FCT: Foot core training
MANOVA: Multivariate analysis of variance
