# Supplementary material for: Effect of adding short foot exercise to hip and knee focused exercises in treatment of patients with patellofemoral pain syndrome: a randomized controlled trial
Source: J Orthop Surg Res. 2024 Apr 1;19:207. doi: 10.1186/s13018-024-04688-x (PMC10983661; doi:10.1186/s13018-024-04688-x)
Supplement: Supplementary file 1 — Supplementary Material 1 [file 13018_2024_4688_MOESM1_ESM.docx]

**APPENDIX : Rehabilitation protocol**

**Guiding principles**

- Dosage is chosen in which the last repetitions are challenging but quality of movement is maintained.
- Dosage individually adjusted once per week by physiotherapist.

**Progression (all exercises):**

- Number of repetitions is increased from 3 sets of 10 repetitions to a maximum of 3 sets of 20 repetitions.
- Thereafter resistance is increased using weight cuff or resistance tubing (see individual exercise).
  - Weight cuffs are available in 0.5 kg increments.
  - Resistance tubing^b^ is selected from 3 possible variants. In order of increasing resistance: red (medium), green (heavy), black (special heavy)

**Other details:**

- Repetitions performed dynamically over 2-3 seconds
- 2-second pause between repetitions.
- 30 second pause between sets.
- Minimum one rest day between sessions

**Hip exercises:**

| **Exercise** | **Position** | **Details** | **Resistance** |
| --- | --- | --- | --- |
| **Hip abduction** | Side-lying | Abduct hip, lifting the straight leg upward. Pelvic stabilization emphasized | Weight cuff fastened at ankle |
| **Hip external rotation (clam-shell)** | Side-lying | Clam-shell position, hip flexed approx. 60°. Pelvic stabilization emphasized | Weight cuff fastened directly below knee. |
| **Hip extension** | Prone | Extend hip, lifting the straight leg upward. | Weight cuff fastened at ankle |

**Knee exercises:**

| **Exercise** | **Position** | **Details** | **Resistance** |
| --- | --- | --- | --- |
| **Straight leg raising** | Supine | Pelvic stabilization emphasized | Weight cuff fastened at ankle |
| **Terminal knee extension** | Supine | Knee supported over a cylinder (ø15cm). Knee extends from 10° to 0°. | Weight cuff fastened at ankle |
| **Mini-squat to 45°** | Standing | Back supported against low-friction wall to reduce stabilizing requirements from hip muscles.  Feet placed shoulder-width apart and 1 foot-length from wall.  Bend knees to 45° | Elastic tubing (length: 2 times distance from lateral femoral epicondyle to medial malleolus) held with one end in each hand, passing beneath both feet. |

**Hott, A., Brox, J. I., Pripp, A. H., Juel, N. G., & Liavaag, S. (2020).** Patellofemoral pain: One year results of a randomized trial comparing hip exercise, knee exercise, or free activity. Scandinavian journal of medicine & science in sports, 30(4), 741-753.

<https://pubmed.ncbi.nlm.nih.gov/31846113/>
